# A Reliable Method of Measuring the Conversion Degrees of Methacrylate Dental Resins

**DOI:** 10.3390/s22062170

**Published:** 2022-03-10

**Authors:** Mirosław Kwaśny, Aneta Bombalska, Karolina Obroniecka

**Affiliations:** 1 Institute of Optoelectronics, Military University of Technology, S. Kaliskiego 2 Str., 00-908 Warsaw, Poland; miroslaw.kwasny@wat.edu.pl; 2 Conservative Dentistry Department, Medical University of Warsaw, S. Binieckiego 6, 02-097 Warsaw, Poland; karolina.obroniecka@wum.edu.pl

**Keywords:** composite resin, degree of conversion, FTIR, LED, halogen, polymerization kinetics

## Abstract

The main aim of the study was to implement the most reliable method of measuring the degrees of conversion during photopolymerization of dental fillings. Contrary to the methods used so far, the method is based only on comparison with the monomer absorbance spectrum without reference bands. Another aim of the study was to prepare a comparative analysis of the polymerization kinetics of dental resins under various light sources and different environmental conditions (irradiance, light dose, temperature), with estimation of the degrees of conversion (DC) of the resins being the main metric. HRi Universal Enamel (UE2) and HRi Universal Dentine (UD2) were examined under two different types of light sources used in dentistry, LED and halogen. DC was measured by Fourier transform infrared spectroscopy (FTIR) in transmission mode from 5 s up to 7 days. Spectra were recorded from the parallel optical layers of samples that were placed between the KBr crystals. The results are expressed by the changes in the absorbance spectrum during the polymerization and the calculated conversion rates. The results of each experiment were averaged from three separate measurements of three samples, during which the samples were illuminated under identical conditions. The data were analyzed by performing ANOVA test comparisons between sample groups at the significance level α = 0.05. The degree of conversion of the UD2 resin was higher than that of UE2 for each experimental condition, but there was no statistically significant difference between the DC of those materials (*p* > 0.05). There was statistically significant difference (*p* < 0.01) in the DC caused by LED and halogen light sources producing the same light doses (38 J/cm^2^). This was the result of different features of light transmission to the filler in the resin composite. The efficacy of the LED source is twice as high as that of the halogen light source. Maximal DC without any other differences in conditions, such as resin type or light source, reached around 70% for temperatures of 22–37 °C. For 37 °C, this took 24 h, which is a contrast to the 7 days it took for 23 °C. The influences of different conditions and factors on reaction kinetics are only strong in the early and the rapid stage of conversion. The optimal time of irradiance using either light source is 20 s for a monolayer, and its thickness should not exceed 2 mm.

## 1. Introduction

Dental resin-based composites (RBCs) are among the most used biomaterials in dentistry and the objects of a very large amount of research—e.g., [1,2,3,4,5,6]. The degree of conversion (DC) of a monomer in a dental RBC is a very important parameter, because the physical and mechanical properties of photo-cured resins are directly influenced by DC [7]. A low DC might cause increased cytotoxicity [8] and reduced hardness. The cured compositions based on the derivatives of methacrylate exhibit considerable numbers of remaining double bonds. It is believed that a limited DC is caused by the mobility of radial chain ends [9]. In general, RBCs reach a DC range from 50–70% [3,4,5], or even 94% [10], depending on the exposure time (or dose), composite composition, temperature, type of photo activator and light source.

Techniques such as Fourier transform infrared spectroscopy (FTIR) [3,4,6,11,12], differential scanning calorimetry (DSC) [13], electron paramagnetic resonance (EPR) [14] nuclear magnetic resonance (NMR) [15], Raman spectroscopy [16,17] and differential thermal analysis (DTA) [18,19], have been used to determine the DC. FTIR spectroscopy is the most frequently used technique but requires certain precautions during sample preparation and testing to obtain an accurate value of DC [4]. Borges et al. [20] carried out a critical review on the conversion degrees of resin monomers by direct analysis. A total of 45 papers were selected, and 15 papers were included in the critical appraisal. Of those 15 papers, 14 evaluated the DC of resin composite monomers using an infrared technique. Apart from the basic requirements for classification of the above studies, such as materials tested, light conditions used and calculations used, the authors took into consideration the acquired spectra. However, absorption spectra are only presented in exceptional cases [6,21]. They are rarely seen in typical spectroscopic analysis.

The current DC determination methods are based on the comparison of the absorption bandwidths at some characteristic peaks, which are typically those representing double-bonded carbon (C=C) monomers and single-bonded carbon reference bands. It is a method that has been used since the 1950s [22], and later it was verified. The most frequently used reference absorption band has a frequency of 1608 cm^−1^, although other frequencies are also used, such as1730 or 4623 cm^−1^ [6]. The method is correct if this reference band shows the same intensity over the entire DC range. This, however, as we have shown, is not accurate for any methyl methacrylate derivative. Even for pure methyl methacrylate, the 1638 and 1608 cm^−1^ bands are not sufficiently separated. This DC calculation method is burdened with an additional, at least 5% systematic error when using a 1608 cm^−1^ frequency band.

We have presented the most reliable method in which no reference bands are needed [23]. It involves studying the kinetics of polymerization of the optical resin layer sandwiched between KBr crystals. The entire cross-section of the light beam from the optical fiber is perpendicular to the crystal’s surface, and the polymerization process of the same sample is measured at any time, from 5 s to even weeks after initiation. The high precision of the method allows us to measure the real effects of the thickness and quality of the irradiated material, minimum exposure times, irradiance amount, irradiance source quality and temperature on the post-polymerization processes. The DC values depend directly on the parameters of the light sources used. Many articles compare the effectiveness of new LED and halogen light sources [24,25]. We have determined the effectiveness of each based on the energy and spectral measurements of the sources, while keeping the unit power of each source and the type of activator the same. Our theoretical analysis was confirmed experimentally, and the experimental results are presented in Section 3, in which we show the dependence of the polymerization rate and DC value depending on the material and the exposure conditions.

The pointwise method of DC measurement does not show the course of a complicated polymerization process. It is a long process consisting of several stages: quick transformation lasting for a few seconds, medium ranging for several minutes and very slow lasting for weeks or months after initiation. Polymerization kinetics studies, although advantageous as compared to in-point measurements, are less prevalent in the literature.

In applied methods, DC is calculated with different levels of accuracy, repeatability and agreement with spectroscopic standards. The differences among those methods are the result of not taking into consideration the time after irradiation. Examples using a kinetic method are the following studies in which the authors used the ATR–FTIR method [3,4,12]. The reflectance method allows one to measure changes in absorbance in layers as thin as several microns due to limited light penetration. In the cited papers, measurement time was restricted only to 5 min after radiation, and as we will show later, post-irradiation processes are significant in DC estimation. 

The authors of the presented paper examined the conversion process in a wider time range after photoactivation. The main goal of this work was to compare polymerization rates of popular dental resin with the use of two classical light sources in different experimental conditions: radiation, exposure time, radiation dose and manner and temperature were varied.

Our preliminary results showed that the maximum values of conversion in the examined resin’s standard conditions (recommended by the manufacturer) do not exceed 70%. Incomplete resin conversion is a very intriguing matter, and explaining it will be significant for dental practice. Our final goal was to get closer to understanding and explaining the conversion process. 

One of the crucial factors in dental resin composites is the presence of fillers, which range from 75–80% of the total content. They restrict light penetration and contact of the monomer with the chains of the polymer. Our polymerization reaction was conducted under different temperatures to allow the expansion of our knowledge about this process. The differences in the kinetics of polymerization in UD2 (Universal Dentine, UD2) and UE2 (Universal Enamel, UE2) confirm the significant influence of the resin filler. As a result, the LED and halogen light results were measured in samples with various thicknesses. 

## 2. Materials and Methods

### 2.1. Resins

The materials used in this study are listed in Table 1. Two resins were used, UE2 and UD2. All experiments were conducted in triplicate.

### 2.2. Measuring Apparatus and Light Sources

(a) Spectrophotometer IS50 ATR, FTIR/FT Raman (Thermo Scientific, Waltham, MA, USA).
(b) Fluorymeter FS 900 (Edinburgh Instr., Livingston, Scotland) for measuring spectra of emission of light sources.
(c) Spectrophotometer Evolution 220 (Thermo Scientific, Waltham, MA, USA) for measuring absorption spectra.
(d) Power meter LaserStar Dual Channel (Ophir Laser Measurement Group, Logan, UT, USA).

We used halogen source Elipar Trilight (ESPE, Seefeld, Germany) and LED (Ledition Polymeryzation Unit, IvocarVivadent, Liechttenstein).

### 2.3. Sample Preparation

**Figure 1:**
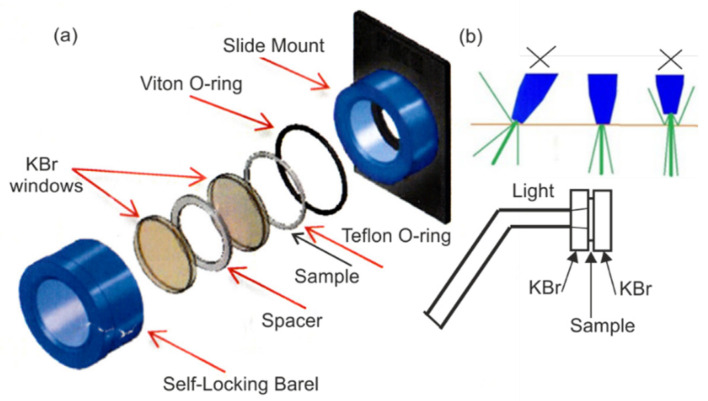
The holder for measuring transmission (**a**). Geometrical arrangement during exposure (**b**).

The samples of resin were placed centrally on the KBr crystal (diameter 25 mm, thickness 3 mm). On the edge of the crystal, a spacer thickness (0.15 mm) and a second KBr crystal were placed. Then, the crystals were slowly pressed to the set thickness in a typical matrix for preparing pellets and then placed in the holder (Figure 1a). The holder for measuring the transmission of the resins and the geometrical arrangement during exposure are shown in Figure 1. The perpendicular incidence of the light beam on the crystal surface and the direct contact between the optical fiber and crystal reduced the amount of reflected radiation. There was light intensity loss due to reflections of about 4%.

Before and after irradiation of the sample with optical fiber (8 mm diameter), the FTIR spectrum was measured at various times. The average spot of light reaching the monomer after passing through the 3 mm crystal was 9 mm, and this was the diaphragm used in a spectrophotometer.

### 2.4. Analysis

**Figure 2:**
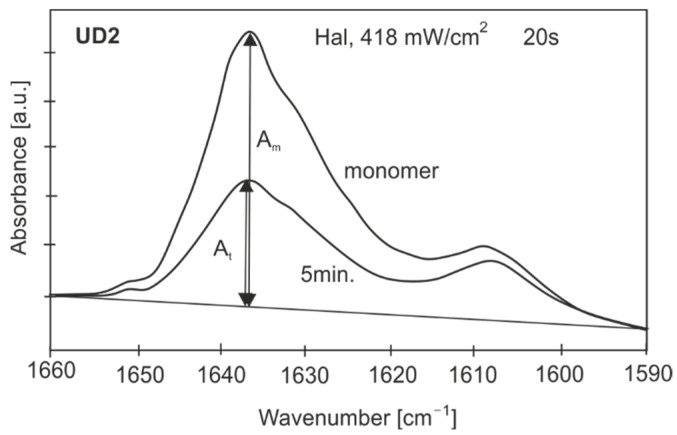
Absorbance changes during polymerization.

To determine the percentage of the remaining unreacted double bonds, the DC was assessed as the variation in the absorbance peak height (intensity) during polymerization (Figure 2).

(1) $$DC\left(t\right)=\left(1-\frac{A_{t}}{A_{m}}\right)\cdot 100\%$$

where *A_t_* is absorbance of a sample in any time periodt, and *A_m_* is the absorbance of the monomer (1638 cm^−1^).

For statistical analysis, the ANOVA method was used. Exemplary results of the statistical analysis are presented in Table 2. 

The confidence interval *(L)* for the probability of *p* = 0.95 is:

(2) $$L=\bar{x}\mp t*s\left(\bar{x}\right)$$

where: *t* is the *t*-Student factor for *p* = 0.95 and *n* =3 (*t* = 3.183). *L* = 54.1 ± 4.55%.

For all experiments, the relative error of measurement was assumed to be ±5%. The presented method is characterized by high precision. The error is a result of the polymerization process. The relative error of absorbance measurements themselves does not exceed 1%.

## 3. Results

### 3.1. Analysis of the Efficiency of Light Sources

**Figure 3:**
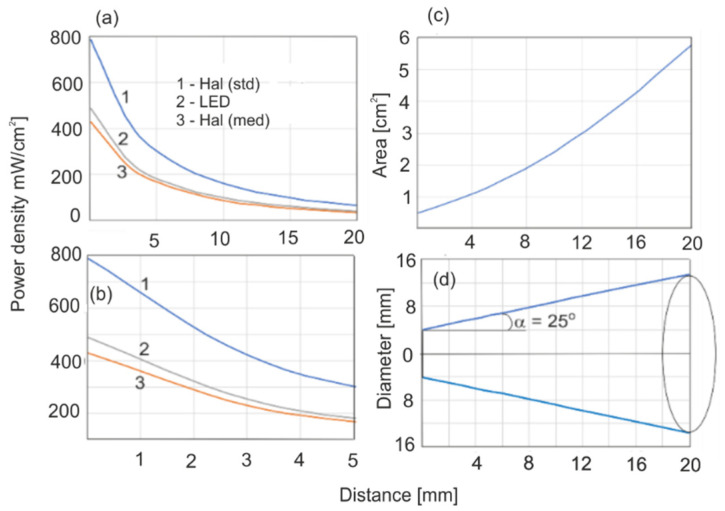
Characteristics of the light sources: irradiance (**a**,**b**), spot surface (**c**), light beam divergence behind the end of the optical fiber (**d**).

Light was supplied to the irradiated surface of the sample with optical fibers. The measured energy characteristics of the sources used are shown in Figure 3. The average irradiance (mW/cm^2^) was determined as the ratio of the average source power to the area of the spot measured. The geometrical conditions during irradiation were chosen so that the tested surface of the sample would coincide exactly with the total cross-section of the beam.

**Figure 4:**
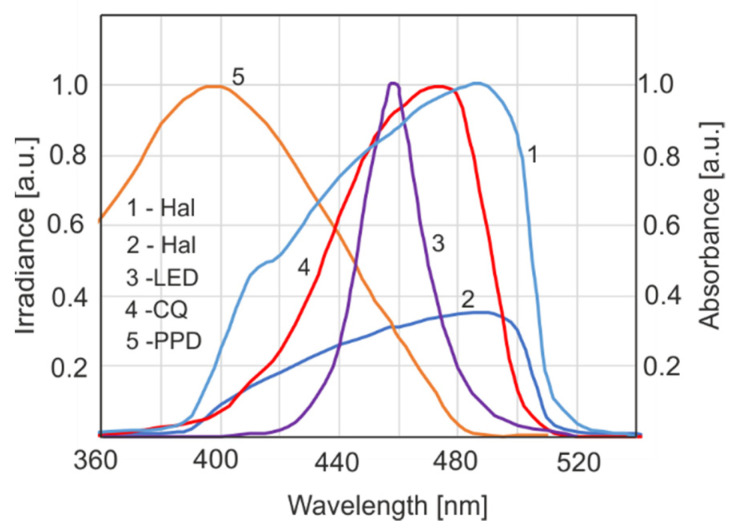
Normalized emission spectra of light sources (1,2,3) and absorption of activators (4,5). The values for curves 2 and 3 are such that the areas represent the same total power.

The efficiency of a light source in the polymerization of dental resin depends on both its spectral characteristics and the locations of the absorption bands of the activator. According to a basic law in photochemistry, only absorbed radiation quanta initiate photochemical processes. The normalized absorption spectra of the activators used—CQ (camphorquinone) and PPD (1-phenyl-1,2 propanedione)—and the normalized emission spectra of light sources are shown in Figure 4.

**Figure 5:**
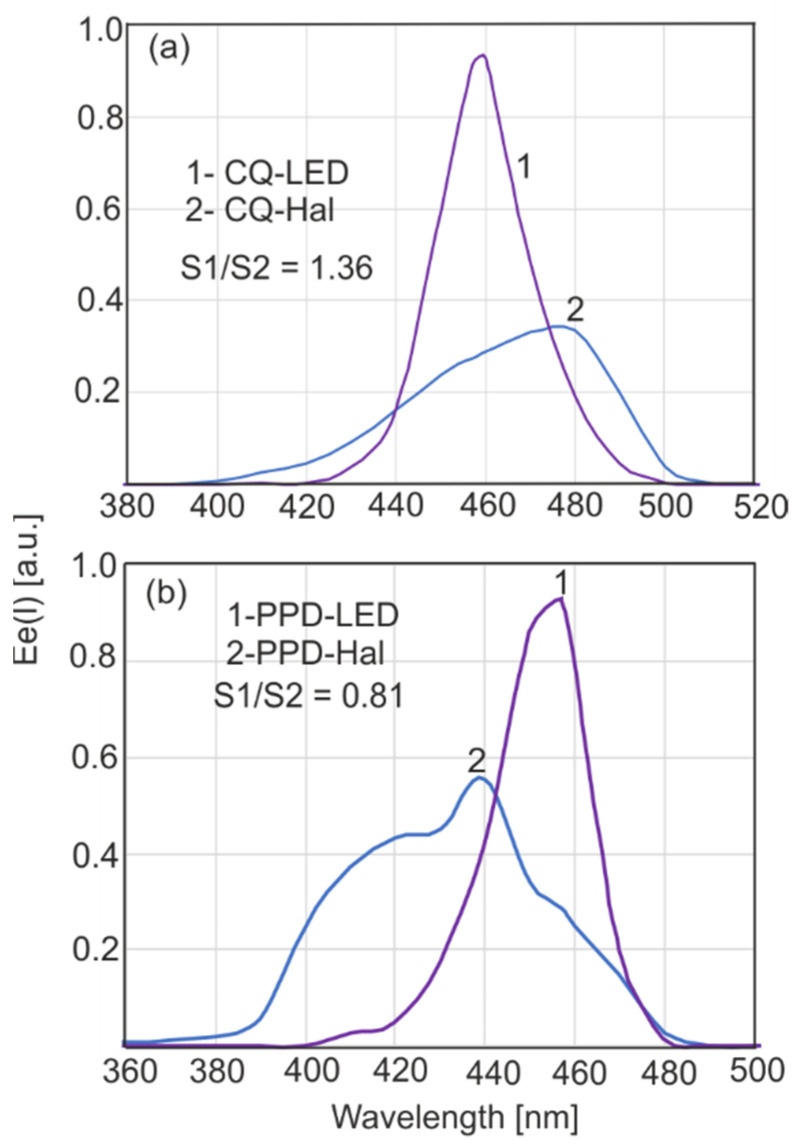
Relative, effective absorption coefficients for CQ (**a**) and PPD (**b**).

Absorption spectra of compounds were determined assuming that for all wavelengths, the intensity of the incident radiation was the same. To compare the actual absorbance of the activator, the emission characteristics of the source should be considered (Figure 5).

The effective total absorption coefficient (*E_e_*) is given by the following:

(3) $$E_{e}=\frac{\int_{\lambda 2}^{\lambda 1}\varepsilon \left(\lambda \right)I\left(\lambda \right)d\lambda }{\int_{\lambda 2}^{\lambda 1}I\left(\lambda \right)d\left(\lambda \right)}$$

where: *ε*(*λ*) is the absorption coefficient of the activator, *I*(*λ*) is the spectral irradiance (mW·cm^−2^·nm) and *λ* is the wavelength.

The ratio of the area under the curves means the relative effectiveness of a source with a particular activator if these sources have the same power or irradiance.

The polymerization conditions of pure resins are significantly different from those of composite materials used in dentistry due to the presence of the fillers, which constitute 75–80% of the composites. The degree of polymerization over time depends on temperature, the emission characteristics of the light source, the irradiance value and light dose and the optical properties of the fillers. 

**Figure 6:**
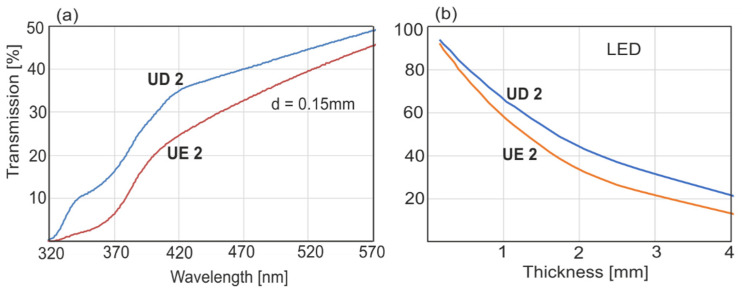
Spectrophotometric characteristics: (**a**) Resin transmission. (**b**) Light transmission with a small distance between the light source (LED, λ = 460 nm) and detector as a function of layer thickness.

Figure 6a shows the transmission spectra of the monomer layer with 0.15 mm thickness measured with a spectrophotometer. The absorption peak of the activator was at around 360–380 nm. Apart from this band, there were no other absorption bands, and the transmission loss was mainly caused by the light scattering effect. Spectrophotometric measurements allow for transmission only towards the direction of light beam propagation. The real values of transmission can be found by measuring the light intensity on the detector placed directly after the sample. In the case of a light scattering material, the mechanism of light transmission is based on multiple light scattering. The results of LED light transmission as a function of resin thickness are presented in Figure 6b.

### 3.2. Measurements of the Conversion Degrees of the Dental Resins

**Figure 7:**
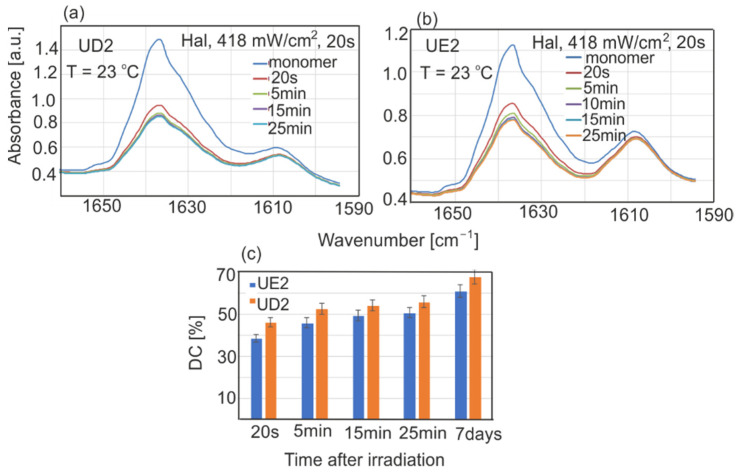
Changes in resin absorption during polymerization after radiation with the halogen (Hal) source at 23 °C: (**a**) UD2, (**b**) UE2, (**c**) calculated DC values.

**Figure 8:**
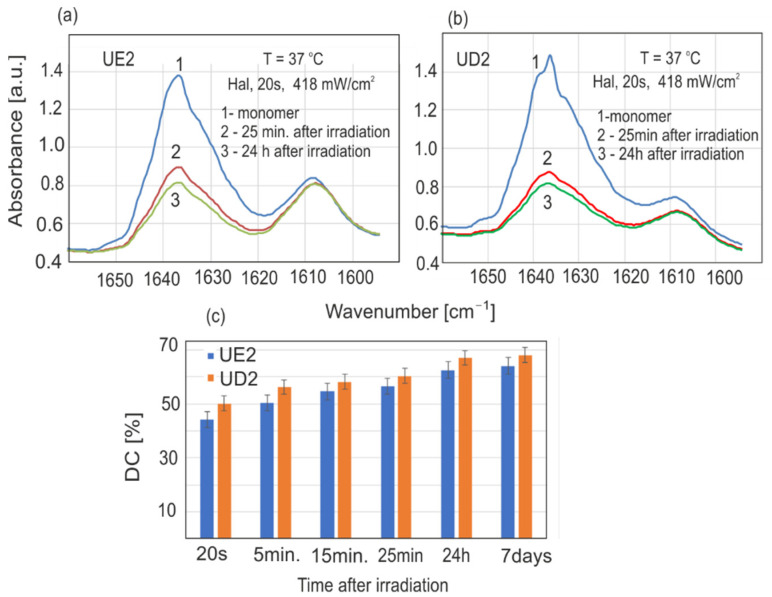
Changes in resin absorption during polymerization after irradiation with a halogen lamp at 37 °C: (**a**) UD2, (**b**) UE2, (**c**) calculated DC values.

**Figure 9:**
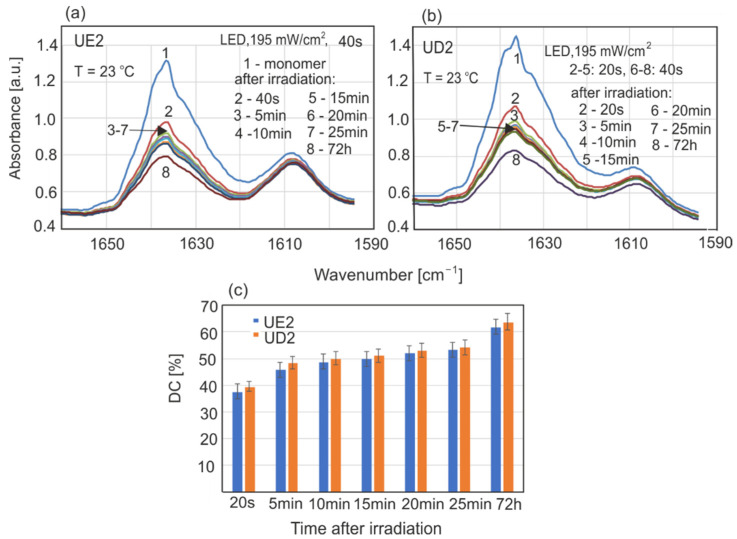
Changes in resin absorption during polymerization after irradiation with an LED at 23 °C: (**a**) UD2, (**b**) UE2, (**c**) calculated DC values.

**Figure 10:**
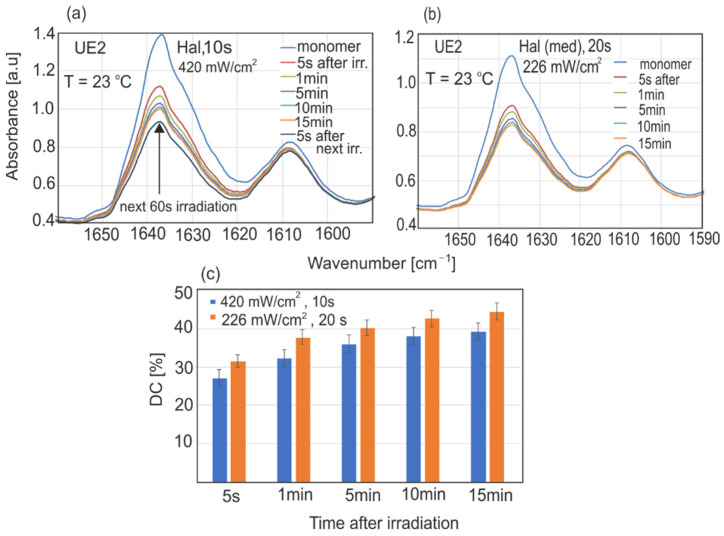
Changes in UE2 resin absorption after LED irradiation with different light doses: (**a**) 10 s with 420 mW/cm^2^, (**b**) 20 s with 226 mW/cm^2^, (**c**) calculated DC values for both variants.

**Figure 11:**
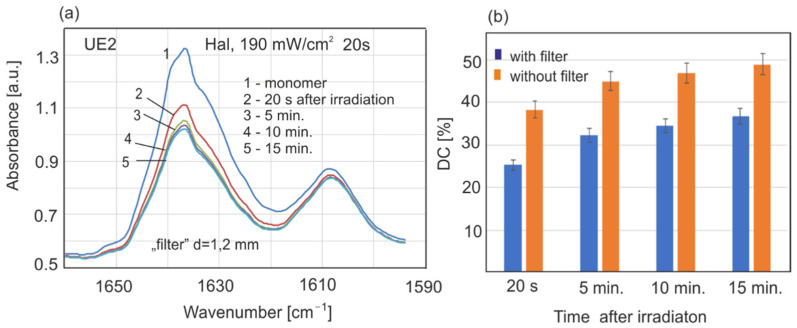
Absorption changes in the UE2 monomer spectrum (**a**), DC of the UE2 irradiated through a polymer ring with 1.2 mm thickness and without a filter (**b**).

The results of polymerization kinetics and the conversion degrees of resins UE2 and UD2 in different experimental conditions are presented in Figure 7, Figure 8, Figure 9, Figure 10 and Figure 11.

The initial exposure time of halogen irradiation was 20 s based on recommendations from the literature [3,4]. The distance between the sample and the optical fiber of 3 mm was chosen as a result of the clinical mandate against overheating the tooth pulp. The halogen light source, although it was equipped with optical filters, emitted some thermal heat, which caused a significant case of preheating. 

The changes in UD2 and UE2 spectra and calculated DC values are shown in Figure 7. The polymerization process starts during irradiation and lasts for a long time, and measurable changes can be visible even after 7 days. The rate of polymerization reached maximal values in the first 5 min after irradiation, and in the next 5–20 min slowed down and leveled off. UD2 showed higher DC values by about 6–8% compared to UE2. After 20 s, UD2 DC values reached approximately 46%, 2/3 of the final value after 7 days (68%).

The vast majority of the experiments carried out were conducted at room temperature (23 °C) due to practical reasons. In such cases, the sample’s temperature before and during measurements was constant. At human body temperature (37 °C), the rate of the polymerization is much faster than at room temperature. The polymerization rates of both resins at 37 °C are shown in Figure 8. One can observe higher DC values for the same time points. Maximal DC values were reached after just 48 h, rather than the 7 days required at 23 °C. 

In the case of the LED lamp, the initial irradiation time was 40 s (Figure 9a). The maximal LED power density was half that of the halogen source. Twice the amount of irradiation time allowed for the application of the same radiation dose. The shortening of LED radiation time to 20 s did not cause significant changes in polymerization rate (Figure 9b). A second irradiation after 15 min also did not cause a significant change in polymerization rate. The chosen irradiation time was therefore appropriate, and with these values, there was no need for extending it.

For halogen source irradiation, 20 s of irradiation time with a power density of 420 mW/cm^2^ seems to be optimal. The time of irradiation and power density were changed to obtain a similar light dose to that provided by the LED. Shortening the irradiation time and power density also caused a significant DC decrease in the first 15 min (Figure 10). 

The minimal thickness of the monomer layer is an important parameter in the polymerization process. The literature data [4] show that different resins possess different optical properties, including depth of light penetration. The presented experimental results were obtained from monomer layers 0.15 mm thick. The drop in transmission through UD2 and UE2 resin layers was small enough that it could be approximated that the light doses received were the same. 

As presented in Figure 3, LED transmission through a polymerized resin ring of UD2 or UE2, 1.2 mm in thickness, was 65 or 54%, respectively. We examined how the monomer layer (UE2 0.15 mm) will behave when irradiated through the polymer ring (1.2 mm), acting as a “filter” (Figure 11). The polymerization rate and recorded DC values are lower than those acquired without the filter, but the polymerization process was still in progress, and after a long period, higher values would have been reached. This is a very important finding. 

**Figure 12:**
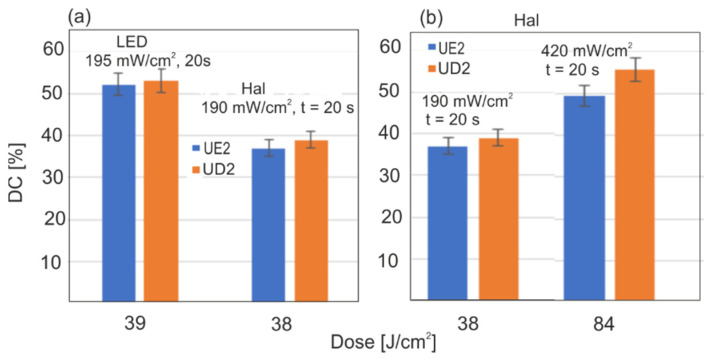
Influences of light source and dose on conversion degree 20 min after irradiation: (**a**) Hal and LED with the same dose; (**b**) different doses with the Hal source.

Another aim of this paper was to compare the photoinitiation efficacies of two light sources: LED and Hal. The results of the light dose’s influence on DC after 15 min of irradiation are shown in Figure 12 (for both light sources). Similar values of DC were obtained when the Hal power density was twice as much as that of the LED. That shows that the LED source was more effective than the halogen one. This is in line with the field effectiveness analysis of the sources presented in Figure 5a.

Methods based on point DC measurements do not show the course of the complicated polymerization process. It is a long-lasting process consisting of several stages: quick transformation lasting few seconds, medium transformation for several minutes and very slow transformation lasting weeks or months after initiation. In the methods applied in other papers so far, DC is calculated with various levels of accuracy and repeatability. The differences between those methods are the results of not taking into consideration, in most cases, the time after irradiation.

In this paper, the polymerization kinetics of two popular dental resins UD2 and UE2 were compared, along with two light sources, LED and Hal. In light of the results, we have come to the conclusion that LED is 40% more effective than Hal. This effectiveness is affected by the optical properties of the filler and light penetration depth. The maximal power density of the halogen source (800 mW/cm^2^) was twice that of the LED (425 mW/cm^2^). This power difference is compensated by the effectiveness of the source, and final DC values were similar for both sources with maximal irradiance parameters. It is worth mentioning that to obtain a similar polymerization rate for both resins, LED power density had to be two times lower than that of the halogen source (Figure 8). In theory, LED efficacy can be heightened. At present, LED sources can have 2–4 W of power density. LEDs do not emit infrared radiation, and illumination of the tooth surface can be performed at a distance of less than 3 mm. In our experiments, we kept a 3 mm distance to provide reproducible conditions for both light sources. As far as Hal is concerned, a 3 mm distance is recommended and a power density of 800 mW/cm^2^ is optimal. In this case, the polymerization rate and DC values can be increased only with a long time of irradiation, but as it was shown, 20 s of irradiation is optimal, and a further dose, even for 60 s, is pointless (Figure 9). According to our analysis of the halogen source’s spectral characteristics, what matters is the lowering of the light dose either by shortening the radiation time (to 10 s) or lowering the power density. Light penetration in the case of the Hal source was the main reason for the limitation of the polymerization rate. This was the reason for the differences in the polymerization rate between UD2 and UE2 (Figure 6 and Figure 7). With the LED, differences in polymerization rate were much smaller, which was a result of the higher spectral irradiance and the greater depth of light penetration as compared to the Hal source. Based on the obtained results, one can conclude that the polymerization rate increases with the power density and light dose. The optimal time of irradiation for a single resin layer is 20 s, and that is in agreement with the literature. The fundamental novelty of our paper is our quantitative approach to finding the long-term polymerization’s influence on final DC values (post-polymerization). The first step of polymerization, during irradiation and within several seconds afterward, is the fastest one, and the DC reaches 50–70% of its maximum value. The remaining phase of polymerization goes slower; it does not end but lasts, despite limited monomer migration. The presented methodology allows one to follow these small changes. In the presented paper, the maximal time of measurement was restricted to 7 days after irradiation; plans for further experiments are in place to monitor the polymerization over months. Despite the polymerization conditions and the rates, the most characteristic feature was that the end DC values stopped at 70%. It is probable that after some time they will increase. The presented results focused on the mechanism and rate of the resin polymerization process. In the literature, this long-term reaction is called the post-polymerization process. It is not a strict description, since the polymerization is still in progress. Classically, polymerization consists of the initiation step, extending the chain and termination. It is noticeable that even with no deliberate irradiance, polymerization continues, and the polymer chains are elongated with subsequent monomers. In the chain-growth of polymers with active radicals, without adding a factor to end the polymerization, the reaction should incorporate the whole monomer volume. In this process, there is no stirring of the monomer volume, and decreased monomer mobility will make the process slow, but does not end it. The question is, for how long is the process extended? To find the answer to this question, one should test the resin polymerization process with no fillers and in higher temperatures.

An important parameter is the estimation of the maximum thickness of the irradiated monomer layer. This thickness depends on the type of filler and the density of light power incident on the surface. For UE2, the LED light power density behind the 1.2 mm thick layer was reduced to 65%, but it was shown that even behind the polymer screening layer, the process was still efficient. UD2 is characterized by higher blue light transmission than UE2, and its maximum layer thicknesses may be higher. The thickness of single UD2 layers recommended by producers for exposure to a halogen source is 1–1.5 mm, which is confirmed by the results of this work. However, these values depend on the power density. The maximum irradiance was 800 (halogen), with a power density of 490 mW/cm^2^ (LED). At a distance of 3 mm from the optical fibers, maximum irradiance and power density decreased to 420 and 195 mW/cm^2^, respectively. Currently, LED sources with much higher power densities are being produced. The recommendations of manufacturers should, in addition to layer thickness and exposure time, include data on power densities.

In practice, the first layer of a monomer should be as thin as possible to maintain a fast polymerization process. Other layers can be thicker and have slower polymerization rates. The optimal time of irradiance for both sources was 20 s for the monolayer. The resin manufacturer’s recommendations are 40 s, but they might be useful for other sources. This confirms how important data linking resin types with irradiance, light dose and film thickness are for a dental practice.

**Figure 13:**
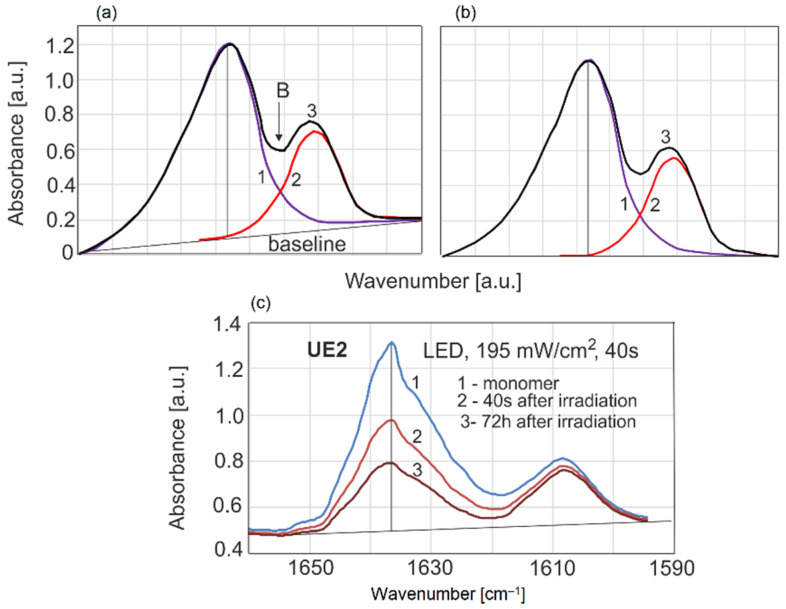
Overlapping of test and reference bands in methacrylate resins in the analytical model: (**a**) with baseline, (**b**) after baseline correction, (**c**) in experimental data.

One of the most important problems is how to calculate DC from spectroscopic measurements. In methods using an internal standard, it is assumed that the ratio of monomer to reference band is constant, that the DC is constant, that the DC depends only on the concentration of monomer or polymer and that this can be used to normalize the thickness of the layer. Figure 13 shows a simulation of two partially overlapping bands and exemplary experimental data. This is a classic case in spectroscopy.

Absorption curve 3 is the arithmetic sum of bands 1 and 2. An inflection point B that is not on the baseline clearly indicates that they are dependent bands. The effect on the mutual intensity of the bands is only at 1608 cm^−1^, but at 1638 cm^−1^, this effect is no longer present. The frequency intensity ratio varies throughout the remaining monomer concentration range; therefore, the errors in the calculation of DC for this reason alone reach over 5%. 

As DC increases, the value of the intensity of the reference band gets closer to the real value, but the value for pure monomer is decisive because it is the reference point for all samples. The courses of curves 1 and 2 can be reproduced with great accuracy mathematically, but that is quite a tedious procedure. A much simpler and more reliable method is to determine the remaining unpolymerized polymer by directly comparing the intensities of the bands at 1638 cm^−1^ and using any reference bands available. This method of determination was presented by Rueggeberg [26]. The authors studied this method to determine the degree of polymerization of polymethyl methacrylate (PMMA) in hexane in the 6165 cm^−1^ band. The procedure is very labor-intensive, as it requires weighing the polymerized substances, extracting the polymers with a solvent and separating the filler from the polymers. Additionally, testing polymerization in real time has already been used, but only with the Attenuated Total Reflection (ATR) method [3,4]. Data from the literature have shown that DC values do not reach 100%. Therefore, we did not know whether achieving complete monomer polymerization would have resulted in the complete disappearance of this band, or whether we were making another inaccuracy at the second basic reference point. The measurements of the absorption of pure commercial PPM layer dispelled these doubts. The completely polymerized polymer does not contain the 1638 cm^−1^ band, which is only characteristic for the monomer.

## 4. Summary

Polymerization of the tested resins consists of three different stages—very fast polymerization from 10–40 s (DC: 25–50%), moderate polymerization (5–25 min, DC: 40–60%) and very slow polymerization (days, weeks, DC: 60–70%). Maximum DC values at 22–37 °C, regardless of polymerization conditions, type of resin and light source, were around 70%. The influences of different conditions and factors on the reaction rate are major only for the initial, fast conversion steps and the time after which the maximum DC value is reached. The increase in DC a long time after activation explains the significant discrepancies in the literature, in which the monitoring time is not specified or varies.

The LED source exhibited greater exposure efficiency compared to halogen source, and for the same DC values, the dose of the LED light was twice as low. The rate of UD2 polymerization is higher than that of UE2, which is caused, among other things, by greater light transmission through the filler. The optimal source irradiance time for a single monomer layer is 20 s, and the thickness of this layer should not exceed 2 mm.

## 5. Conclusions

We presented a reliable method of determining the degree of polymerization of a dental resin at any time after exposure, and used two resins for experimental validation.

The method allows for an accurate comparison of the polymerization capacities of different materials. Factors influencing correct measurement were presented. For example, the influence of the distance of the optical fiber from the sample under irradiance was considered. FTIR spectra of the surface area of each sample as it was irradiated were measured, which eliminated the lack of uniformity of the optical fiber irradiation.

It was confirmed by experimentation that an LED source is more efficient than a halogen one in the case of a CQ activator.

## Figures and Tables

**Table 1:** Information about using composite materials.

Materials	Components
Enamel Plus HR*i* Universal Enamel (UE2), GDF GmbH 61191—Rosbach, Germany	Monomers (20%) Diurethandimethacrylate Iso-propyliden-bis (2(3)-hydroxy-3(2)-4 (phenoxy)propyl-bis(methacrylate) (Bis-GMA) 4-Butandialdimethacrylate Filler (80%)—Glass filler (68%), average particle size 1.0 mm, Zirconia (12%), particle size 20 nm Activator—camphorquinone (CQ)
Enamel Plus HR*i* Universal Dentine (UD2), GDF GmbH 61191—Rosbach, Germany	Monomers (25%)—the same composition. Filler (75%)—Glass filler, medium particle size 0.7µm, silicon dioxide, medium size—0.04 µm Activator—camphorquinone (CQ)

**Table 2:** Exemplary statistical analysis based some results.

DC_i_ [%] (*x*)	$\bar{x}$	SD (s) [%]	$s\;(\bar{x})$ [%]
54.1 56.2 52.1 55.7 53.3	54.3	1.69	0.976

## Data Availability

The study did not report any data.
